# Unions and employment of migrant workers in China: a causal analysis using the treatment effect model

**DOI:** 10.3389/fpsyg.2026.1770532

**Published:** 2026-02-18

**Authors:** Zirui Guo, Mingchen Yang, Hao Sun

**Affiliations:** College of Economics and Management, Shanghai Ocean University, Shanghai, China

**Keywords:** employment effect, migrant workers, socio-economic integration, treatment effect model, union participation

## Abstract

Migrant workers in China often experience employment instability due to limited labor protection and restricted access to formal institutions, which undermines both job security and social integration. Although trade unions are expected to support workers’ employment, their role remains debated in the Chinese context. Using pooled data from the China General Social Survey (2010–2021), this study examines the effect of union participation on migrant workers’ employment by applying a treatment effect model. The results indicate that union membership is significantly associated with a higher likelihood of non-agricultural employment. Mechanism analyses suggest that unions improve employment outcomes by enhancing labor rights protection, promoting formal contracts, and expanding social insurance coverage, thereby reducing perceived employment uncertainty and strengthening labor market attachment. These findings suggest that union participation is associated with improved employment outcomes, potentially through institutional and psychological pathways. However, it is important to acknowledge that unions may also serve as markers of pre-existing formalized employment environments.

## 1 Introduction

Employment stability is a fundamental determinant of individuals’ psychological well-being, social integration, and long-term life satisfaction. Research in psychology and organizational behavior has consistently shown that job insecurity is associated with increased anxiety, stress, and reduced subjective well-being (Sönmez et al., 2023; Murphy and Turner, 2023). Stable employment not only provides economic security but also contributes to a sense of predictability, control, and social belonging, which are essential for psychological functioning. For migrant workers, who often occupy disadvantaged positions in segmented labor markets, employment instability poses particularly severe challenges to both economic security and mental well-being.

In China, migrant workers face additional institutional constraints stemming from the household registration system and unequal access to formal labor protection. These constraints increase their likelihood of informal employment, weak contract enforcement, and limited social insurance coverage. As a result, migrant workers are more exposed to employment uncertainty and labor market risks than urban residents. From an interdisciplinary perspective, understanding how institutional arrangements can mitigate employment instability among migrant workers is critical for linking labor market outcomes with psychological security and social integration (Zhang et al., 2022).

A substantial body of literature has examined the role of trade unions in shaping labor market outcomes. Classical labor economics highlights that unions influence wages, working conditions, and labor relations through collective bargaining and institutional voice mechanisms (Alexiou and Trachanas, 2023). Empirical studies in both developed and developing countries have documented union effects on wages, income inequality, and workers’ welfare. In the Chinese context, existing research has focused primarily on the impact of unions on wages, working hours, labor contract coverage, and compliance with labor regulations (Booth et al., 2022). These studies provide important insights into the economic functions of trade unions within China’s evolving labor market institutions.

Despite these contributions, several limitations remain in the current literature. First, most studies emphasize wage-related outcomes, while relatively little attention has been paid to employment outcomes and job stability. For migrant workers, employment stability may be more salient than wage levels, as it directly affects perceived job security and psychological well-being. Second, many empirical studies rely on firm-level or regional data, which may limit the generalizability of their findings to the broader migrant worker population. Third, few studies explicitly explore the mechanisms through which trade unions may reduce employment uncertainty and enhance workers’ attachment to the labor market, particularly from a behavioral or psychological perspective (Sayre, 2023).

This study aims to address these gaps by examining the employment effects of trade union participation among migrant workers in China. The present research makes three main contributions. First, it shifts the focus from wage effects to employment outcomes, thereby highlighting a dimension of labor market performance that is closely linked to psychological security and social integration. Second, using nationally representative survey data, this study provides causal evidence on the impact of union participation that extends beyond localized or firm-specific contexts. Third, through mechanism analysis, this study elucidates how trade unions function as institutional support and risk-sharing arrangements by improving labor rights protection, promoting formal labor contracts, and expanding access to social insurance.

Using pooled data from the China General Social Survey (2010–2021), this study employs a treatment effect model to estimate the causal impact of union participation on migrant workers’ employment outcomes. The empirical results indicate that union membership significantly increases the likelihood of non-agricultural employment. Further analyses suggest that this effect operates through enhanced labor rights enforcement, greater contract formalization, and broader social insurance coverage, which collectively reduce employment uncertainty and strengthen labor market attachment. By linking institutional arrangements to employment stability, this study contributes to the interdisciplinary literature on work, institutions, and well-being, and offers practical implications for policies aimed at improving employment stability among vulnerable worker groups.

## 2 Theoretical analysis and research hypotheses

Trade unions, as collective organizations representing workers’ interests, play an important role in labor markets through collective bargaining, labor rights protection, and the provision of social welfare. Classical labor economics emphasizes that unions influence labor market outcomes by correcting power imbalances between employers and workers and by institutionalizing workers’ collective voice (Biasi and Sarsons, 2022). From the perspective of internal labor market theory and employment protection theory, unions can reduce employment uncertainty by stabilizing employment relationships, standardizing labor practices, and expanding access to social security (Chadi and Goerke, 2023).

However, unions may also exhibit monopolistic characteristics, potentially generating negative externalities in the labor market (Freeman and Medoff, 1979). In practice, the net effect of unions depends on institutional context. In China, trade unions assume a dual role as both defenders of workers’ rights and stabilizers of social order (Lei, 2024). Although unions are not fully autonomous from local governments, their emphasis on social stability and labor protection has substantially weakened their monopolistic tendencies and strengthened their role in safeguarding workers’ rights (Clarke and Sahin-Dikmen, 2020). Within this institutional framework, unions are expected to play a particularly important role for migrant workers, who are typically characterized by weak bargaining power, informal employment, and limited access to social protection.

Based on established theoretical foundations and existing empirical evidence, this study conceptualizes three clearly defined and interrelated pathways through which union participation may affect migrant workers’ employment outcomes. These pathways are (1) labor rights protection and work-hour regulation, (2) employment relationship formalization through labor contracts, and (3) social security coverage and labor market attachment. Importantly, these pathways are consistent with and empirically tested in the subsequent mechanism analysis.

### 2.1 Labor rights protection and work-hour regulation

First, unions may improve migrant workers’ employment outcomes by enhancing labor rights protection and regulating working hours. Migrant workers generally occupy a disadvantaged position in segmented urban labor markets, often facing excessive working hours, high labor intensity, and weak enforcement of labor standards (Xue and Li, 2022). According to collective bargaining theory, unions can negotiate with employers to establish more reasonable work-hour arrangements and reduce excessive overtime, thereby improving working conditions (Freeman and Medoff, 1979).

In the absence of unions, firms may extend working hours and intensify labor input to reduce unit labor costs, which can lead to worker exhaustion, higher turnover, and reduced long-term employment stability (Sönmez et al., 2023). By contrast, unions can promote the implementation of standard work-hour systems and limit excessive labor intensity through collective bargaining and workplace supervision (Blanchflower and Bryson, 2025). Reduced working hours not only alleviate physical strain but also contribute to higher labor productivity per unit of time and lower job separation rates (Gihleb et al., 2024). From an employment perspective, the regulation of working hours can stabilize labor supply and improve workers’ ability to remain continuously employed.

Therefore, by protecting basic labor rights and standardizing working hours, unions can enhance employment stability and improve migrant workers’ attachment to the labor market.

### 2.2 Employment relationship formalization through labor contracts

Second, unions may affect migrant workers’ employment by promoting the formalization of employment relationships, particularly through increasing labor contract coverage. Employment protection theory emphasizes that formal labor contracts reduce uncertainty by clearly defining rights and obligations for both workers and employers (Ferreiro and Gomez, 2020). In China, many migrant workers remain employed without written contracts, which exposes them to arbitrary dismissal, wage arrears, and unstable employment (Ma et al., 2025).

Unions can encourage and supervise the signing of labor contracts through collective bargaining and rights protection mechanisms (Booth et al., 2022). The existence of labor contracts strengthens job security by reducing informal employment and stabilizing employe–employee relationships. Empirical studies have shown that workers with formal contracts experience lower job turnover and higher employment stability.

For migrant workers, labor contract formalization not only reduces employment uncertainty but also provides an institutional basis for accessing other employment-related benefits, such as social insurance and legal protection (Aizawa et al., 2024). By promoting labor contract signing, unions help migrant workers establish longer-term and more stable employment relationships, thereby improving overall employment outcomes.

### 2.3 Social security coverage and labor market attachment

Third, unions may improve migrant workers’ employment by expanding access to social security, particularly health insurance, thereby strengthening workers’ labor market attachment. From the perspective of social risk-sharing theory, social insurance reduces workers’ vulnerability to income shocks caused by illness or injury, which in turn affects labor supply decisions and employment continuity (Yixin et al., 2025).

Migrant workers are often excluded from urban social security systems due to informal employment arrangements and employer non-compliance. Unions can play an important role in promoting employers’ fulfillment of social insurance obligations and in raising workers’ awareness of their social security rights (Cheng and Wei, 2024). Health insurance coverage, in particular, reduces the economic burden of illness and lowers the risk of forced labor market exit due to health shocks (Wang et al., 2023).

Improved social security coverage enhances migrant workers’ expectations of future income and reduces perceived employment risk, thereby increasing their willingness to remain employed and stay in urban labor markets (Aizawa et al., 2024). In this sense, unions contribute to employment stability not only through direct labor market interventions but also by strengthening workers’ long-term labor market attachment.

### 2.4 Research hypotheses

Based on the above theoretical analysis, this study proposes the following hypotheses:

*H1*: Other things being equal, union participation significantly improves migrant workers' employment.

*H2*: Unions play a positive role in migrant workers’ employment through three pathways: enhancing labor rights protection, increasing the stability of employment contracts, and promoting urban social integration.

## 3 Data and methodology

### 3.1 Data source and sample construction

The primary data for this study is derived from the China General Social Survey (CGSS) database and the China Statistical Yearbook. The CGSS database provides nationwide, comprehensive, and continuous micro-level survey data, which offers valuable support for tracking changes in institutional, structural, behavioral, and attitudinal trends, as well as the evolving roles, statuses, and perceptions of social members and groups. Additionally, the database provides rich micro-level data regarding migrant workers’ participation in trade unions. Based on this, the study selects data from the years 2010, 2011, 2012, 2013, 2015, 2017, 2018, and 2021 as the research sample.

Based on the research theme, this study employs a multi-dimensional approach to systematically clean the raw data. First, in consideration of the coverage characteristics of trade union organizations in China and the specificities of the migrant worker group, the sample is restricted to workers who meet all the following criteria: (1) registered as agricultural household (hukou) holders; (2) employed in non-agricultural industries; (3) employed status (excluding employers and self-employed individuals). Second, the sample boundaries are defined according to the legal working age, with male participants aged 16–60 and female participants aged 16–55. Finally, invalid samples are excluded based on the presence of missing key variables (e.g., unreported annual income) or logical anomalies (e.g., negative working hours).

After the standardization process, the final dataset consists of a pooled cross-sectional data with 13,347 observations. The union coverage rate in the sample is 10.64%, a figure comparable to the national union membership rate among migrant workers based on macro-level statistics. From a temporal perspective, the distribution of the sample across the survey years is as follows: 2010 (1,515), 2011 (855), 2012 (1,718), 2013 (1,836), 2015 (1,702), 2017 (2,127), 2018 (2,237), and 2021 (1,357). It should be noted that data for the years 2014, 2016, and 2019–2020 is missing due to the absence of surveys or unpublished data. This intermittent feature will be controlled for in the subsequent analysis using time fixed effects.

### 3.2 Variable definitions and descriptive statistics

#### 3.2.1 Dependent variable

The employment status of migrant workers ( ${\mathrm{Emp}}_{i}$ ) is defined as follows: if the worker is engaged in non-agricultural employment, the value of ${\mathrm{Emp}}_{i}$ is 1; if the worker is unemployed, the value is 0.

#### 3.2.2 Core independent variable

The variable ${\text{union}}_{i}$ indicates whether the worker is a union member. If the worker is a union member, the value of union is 1; otherwise, it is 0. Additionally, the model includes a series of control variables for individual characteristics and city-level characteristics to minimize the potential impact of omitted variable bias on the results. These control variables are included to account for factors that might otherwise influence the relationship between union membership and the outcomes of interest.

#### 3.2.3 Control variables

The individual-level control variables include Age (*age*) and its square term (*age^2^*): The increase in a worker’s years of employment is generally accompanied by the accumulation of work experience. However, with age, the ability to update skills and adapt to new tasks may gradually decline. Therefore, the square term of age is introduced to capture this nonlinear relationship. Gender (*gender*): Gender differences may lead to significant disparities in how male and female workers adapt to production tasks, career choices, and income levels. In this study, male workers are assigned a value of 1, and female workers are assigned a value of 0. Marital status (*marriage*): Marital status can affect a worker’s employment decisions and career planning. Married workers often bear more family responsibilities. Therefore, married individuals are assigned a value of 1, while other statuses (e.g., single, divorced) are assigned a value of 0. Skill level (*skill*): A worker’s skill level is an important factor influencing their employment. In this study, skill levels are categorized based on educational attainment: low skill (junior high school or below, assigned a value of 1), medium skill (high school/vocational school or associate degree, assigned a value of 2), and high skill (bachelor’s degree or above, assigned a value of 3).

The city-level control variables include the following economic and structural indicators: Economic development level (*lnagdp*): Measured as the logarithm of per capita GDP. Industrial structure (*industry*): Measured by the ratio of the added value of the secondary and tertiary industries to GDP. Consumption level (*consume*): Measured as the ratio of total urban household consumption to regional GDP. Average wage level (*lnawage*): The logarithm of the average wage of urban employees is used as a reference indicator for the overall income level of workers.

In addition, the model includes province dummy variables and year dummy variables to control for fixed effects at the regional and temporal levels, thus eliminating the influence of unobservable regional factors and time trends on the results. The inclusion of these control variables helps provide a more comprehensive understanding of the mechanism through which trade unions affect migrant workers’ employment. The definitions and descriptive statistics of the main variables in this study are presented in Table 1.

**Table 1:** Definitions of key variables and descriptive statistics results.

Variable	Definition	Overall	In union	Not in union
*Emp*	Non-farm employment = 1, Unemployed = 0	0.808 (0.393)	0.940 (0.237)	0.802 (0.398)
*income*	The logarithm of annual income	9.946 (1.053)	10.399(0.977)	9.925 (1.052)
*age*	Survey year–Birth year	39.299 (10.889)	39.597 (10.255)	39.284 (10.918)
*gender*	Female = 0, Male = 1	0.560 (0.496)	0.641 (0.480)	0.556 (0.497)
*marriage*	Not married = 0, Married = 1	0.819 (0.385)	0.840 (0.367)	0.818 (0.386)
*skill*	Junior high school or below = 1, High school/Secondary vocational school and junior college = 2, Bachelor’s degree or above = 3	1.343 (0.559)	1.740 (0.698)	1.324 (0.544)
*lnagdp*	The logarithm of per capita GDP (in yuan)	10.767 (0.449)	10.851 (0.493)	10.763 (0.446)
*industry*	The proportion of the added value of the secondary and tertiary industries in GDP (%)	90.493 (4.786)	91.617 (4.890)	90.439 (4.774)
*consume*	The ratio of urban residents’ consumption to regional GDP (%)	28.050 (4.838)	28.059 (4.321)	28.050 (4.861)
*lnawage*	The logarithm of the average wage of urban employees (in yuan)	10.963 (0.323)	11.011 (0.351)	10.960 (0.321)

The numbers outside the brackets in the table represent the mean, and the numbers inside the brackets represent the standard deviation.

### 3.3 Econometric model and identification strategy

To examine the relationship between union participation and migrant workers’ employment, we first estimate a baseline model using Ordinary Least Squares (OLS) regression, which provides an initial assessment of the association while controlling for observable confounders (Equation 1):


$$\mathrm{Em}p_{i}=\alpha +\beta_{1}\text{unio}n_{i}+\gamma_{1}X_{1}+\gamma_{2}X_{2}+\varepsilon_{i}$$ (1)

In this model, the dependent variable ${\mathrm{Emp}}_{i}\;$ represents the employment status of migrant workers. ${\text{union}}_{i}\;$ denotes the union participation variable, indicating whether the individual is a union member. $X_{1}$ and $X_{2}$ represent individual-level and regional-level control variables for migrant workers, respectively, and $\varepsilon_{i}$ is the error term.

However, OLS estimates are likely biased due to endogeneity stemming from self-selection. Union membership is not randomly assigned; it reflects a conscious decision influenced by psychological, social, and contextual factors that are often unobserved. Migrant workers who join unions may differ systematically from non-members in ways not captured by standard controls, such as: (1) Psychological traits: higher risk tolerance, stronger future orientation, greater trust in collective institutions; (2) Social and cognitive resources: broader informal networks, greater awareness of labor rights, enhanced political efficacy; (3) Situational factors: employment in firms with more formalized HR practices or a culture of compliance.

These unobserved characteristics likely influence both the propensity to unionize and employment outcomes, creating a classic case of selection bias. Without addressing this, the estimated “effect “of union membership may merely reflect these underlying differences—a dressed-up correlation rather than a causal relationship.

To address self-selection and omitted variable bias and to identify the causal effect of union participation, we employ a Treatment Effect Model as our primary identification strategy. Following Maddala (1983) for handling binary endogenous regressors, we specify a two-equation system:


$$D=\eta +\mathrm{\lambda Z}+\zeta_{1}X_{1}+\zeta_{2}X_{2}+\zeta_{3}X_{3}+\zeta_{i}$$ (2)

In this model, if D≥0 , then ${\text{union}}_{i}=1$ otherwise ${\text{union}}_{i}=0$ . Z is an independent explanatory variable. Based on the study by Cui et al. (2022), we employ provincial-level data from the China Labor Statistical Yearbook to construct a set of instrumental variables for union involvement. Specifically, these include: (1) the ratio of labor dispute cases mediated by trade unions to the total number of labor disputes in the province; (2) the ratio of union-established training institutions to the total number of union organizations in the province; and (3) the ratio of unions that organize labor competitions to the total number of union organizations. In Equation (3), $X_{3}$ represents this vector of instrumental variables, originally used in the selection equation (Equation 2). These variables are hypothesized to influence an individual’s probability of union membership while remaining exogenous to the error term in the outcome equation, thereby addressing potential endogeneity in estimating the effect of union participation on employment outcomes.

To simplify the analysis, we assume that the disturbance terms $(\varepsilon_{i},\zeta_{i})\sim N(0,0,\sigma^{2},1,\rho ),$ where ρ represents the correlation coefficient between $\varepsilon_{i}\;$ and $\zeta_{i}$ . If ρ≠0 , this indicates the presence of endogeneity in the econometric model. Ω is the inverse Mills ratio, defined as $\Omega (c)=\frac{\phi (c)}{1-\phi (c)},$ where *ϕ*(c) is the cumulative distribution function of the standard normal distribution. The final estimation equation is as follows:


$$\begin{array}{l} E(\mathrm{Em}p_{i}|\text{unio}n_{i}=j,X_{1},X_{2},X_{3})=\alpha +\text{βunio}n_{i}+\gamma_{1}X_{1}+\gamma_{2}X_{2}+\\ \gamma_{3}X_{3}+\rho \sigma \Omega_{i}+\varepsilon_{i},j=0\;\text{or}\;1 \end{array}$$ (3)

The treatment effect model relies on the assumption that selection into union membership is jointly determined by observable individual characteristics and exogenous variation in the institutional environment captured by the selection equation. Conceptually, the included covariates proxy migrant workers’ access to information, employment formality, and exposure to collective institutions, which are central channels shaping union participation in China. The provincial-level union activity indicators are intended to reflect institutional capacity and organizational penetration that affect the likelihood of union membership rather than individual employment outcomes directly. We acknowledge that regional labor market institutions may influence employment through multiple channels; therefore, province and year fixed effects, as well as city-level labor market controls, are included to absorb systematic regional heterogeneity. While the exclusion restriction cannot be directly tested, the consistency of results across alternative specifications and matching-based approaches provides supporting evidence that the estimated effects are not solely driven by unobserved selection. Accordingly, we interpret the findings as indicating a plausibly causal employment effect of union participation, rather than definitive causal proof.

## 4 Regression results

### 4.1 Baseline regression

Table 2 presents the baseline regression results. Columns (1) through (3) report the estimated associations between union membership and migrant workers’ employment under progressively richer sets of control variables. The results indicate a positive and statistically significant association, providing initial support for Hypothesis H1. However, it is crucial to interpret these OLS estimates as conditional correlations, not as causal effects. The positive coefficient may reflect the influence of unobserved factors, such as workers selecting into unionized firms that offer inherently better employment conditions, or firms with more formalized human resource practices being more likely to host union branches. While we control for a range of individual and regional characteristics, the possibility of omitted variable bias remains.

**Table 2:** Baseline regression results.

Variable	Emp		
	(1)	(2)	(3)
*Union*	0.129^***^ (0.014)	0.085^***^ (0.012)	0.086^***^ (0.012)
*Age*		0.036^***^ (0.004)	0.036^***^ (0.004)
*Age2*		−0.001^***^ (0.000)	−0.001^***^ (0.000)
*gender*		0.132^***^ (0.010)	0.132^***^ (0.010)
*marriage*		0.018 (0.014)	0.018 (0.014)
*skill*		0.065^***^ (0.007)	0.065^***^ (0.007)
*lnagdp*			0.287 (0.314)
*industry*			−0.011 (0.015)
*consume*			0.007 (0.009)
*lnawage*			−0.263 (0.317)
Time fixed effect	Yes	Yes	Yes
Province fixed effect	Yes	Yes	Yes
*N*	13,347	13,347	13,347
*R* ^2^	0.183	0.866	0.866

① ^***^, ^**^, and ^*^ represent significance levels of 1, 5, and 10%, respectively. ② The numbers in parentheses are robust standard errors clustered at the provincial level.

### 4.2 Addressing endogeneity: treatment effect estimates

To credibly identify the causal impact of union membership, this section presents the core results from the Treatment Effect Model (TEM), which is specifically designed to correct for endogeneity. The estimation methods for treatment effects primarily include Maximum Likelihood Estimation (MLE) and Two-Stage Estimation (TS). The regression results for both methods are presented and compared with the OLS regression results, as shown in Table 3.

**Table 3:** Endogeneity treatment results.

Variable	Emp		
	(1)	(2)	(3)
	OLS	TEM-TS	TEM-MLE
*Union*	0.086^***^ (0.012)	0.861^***^ (0.320)	0.571^***^ (0.011)
Control variables	Yes	Yes	Yes
Time fixed effect	Yes	Yes	Yes
Province fixed effect	Yes	Yes	Yes
H_0_: ρ=0			5,012.01***
*N*	13,347	13,347	13,347
*R* ^2^	0.183	0.866	0.866

① ^***^, ^**^, and ^*^ represent significance levels of 1, 5, and 10%, respectively. ② The numbers in parentheses are robust standard errors clustered at the provincial level.

Table 3 presents the estimation results. Columns (1) through (3) display the results for the relationship between union membership and migrant workers’ employment, estimated using OLS, the two-stage treatment effect model (TEM-TS), and the maximum likelihood treatment effect model (TEM-MLE), respectively. The rejection of the null hypothesis at the 1% level in Column (3) confirms the presence of endogeneity, justifying the use of the treatment effect model. The statistically significant coefficients in Columns (2) and (3) indicate a positive relationship between union membership and employment outcomes after addressing endogeneity concerns. However, these estimates should be interpreted as model-dependent associations rather than definitive structural effects. An important alternative explanation is that union membership may serve as a proxy for employment in larger, more regulated firms that inherently offer better working conditions. While our identification strategy attempts to isolate the effect of union participation, we acknowledge that our estimates may partially reflect this underlying institutional context. Following methodological recommendations (Li and Xu, 2014), subsequent analyses report results based on the more efficient MLE estimator.

### 4.3 Robustness checks

Having established a robust causal relationship using the TEM, we proceed to conduct a series of supplementary analyses to assess the sensitivity of our findings to different model specifications and measurement approaches.

#### 4.3.1 Alternative model specifications

Given that migrant workers’ employment is a binary variable, the Probit and Logit models, which more accurately capture the distributional characteristics of binary variables, provide more reliable estimates. Therefore, this study re-estimates the regression results using both the Probit and Logit models. Columns (1) and (2) of Table 4 present the regression results from these models. Consistent with the baseline regression results, this suggests that the findings in this study are robust.

**Table 4:** Robust test.

Variable	Emp			
	(1)	(2)	(3)	(4)
	Probit	Logit	PSM	Replace the Core Explanatory Variable
*Union*	0.584^***^ (0.093)	1.099^***^ (0.183)	0.075^***^ (0.016)	
*Union_2*				0.234^**^ (0.108)
Control variables	Yes	Yes	Yes	Yes
Time fixed effect	Yes	Yes	Yes	Yes
Province fixed effect	Yes	Yes	Yes	Yes
*N*	13,347	13,347	2,315	13,347
*R* ^2^	0.121	0.068	0.302	0.371

① ^***^, ^**^, and ^*^ represent significance levels of 1, 5, and 10%, respectively. ② The numbers in parentheses are robust standard errors clustered at the provincial level.

#### 4.3.2 Propensity score matching

Given the potential selection bias between union membership and migrant workers’ employment, the Propensity Score Matching (PSM) method serves as an effective tool to address this issue. To more accurately assess the impact of unions on migrant workers’ employment, this study employs the PSM method to match union members and non-members with similar characteristics, thereby reducing the interference of selection bias. Specifically, the Logit model is first used to calculate the propensity scores while controlling for factors such as gender, age, skill level, marital status, province, and year. Then, based on the propensity scores of union members and non-members, a 1-to-1 nearest neighbor matching method is applied with a caliper of 0.05. The regression results in Column (3) of Table 4 show that the positive effect of unions on migrant workers’ employment remains significant at the 1% level, demonstrating that, after controlling for selection bias, unions still significantly promote migrant workers’ employment and increase their income.

#### 4.3.3 Alternative measures of union presence

Simply using whether migrant workers join a union as an explanatory variable may not fully capture the comprehensive impact of unions on employment, as it overlooks the specific functions of unions and their role in different regional labor markets. Therefore, this study further introduces the total number of provincial-level union organizations from the China Labor Statistical Yearbook as a proxy variable for union functions (*Union_2*), to more comprehensively examine the impact of unions on migrant workers’ employment. The regression results presented in Columns (4) and (5) of Table 4 show that the total number of provincial-level union organizations has a significant positive impact on migrant workers’ employment and income. This suggests that the broader functional role of unions can effectively promote migrant workers’ employment.

### 4.4 Heterogeneity analysis

While the average treatment effect provides an overall picture, the impact of union membership may vary across different subgroups of migrant workers. To explore this, we conducted subgroup analyses based on skill level, gender, and marital status. The results, presented in Table 5, reveal meaningful heterogeneity in both statistical significance and economic magnitude.

**Table 5:** Heterogeneity analysis.

Variable	Emp						
	Group according to skill			Group according to gender		Group according to marriage status	
	Low-skilled	Mid-skilled	High-skilled	Male	Female	Not in marriage	In marriage
	(1)	(2)	(3)	(4)	(5)	(6)	(7)
*Union*	0.089^***^ (0.021)	0.070^***^ (0.015)	0.123^***^ (0.026)	0.062^***^ (0.012)	0.136^***^ (0.023)	0.115^***^ (0.028)	0.082^***^ (0.011)
Control variables	Yes	Yes	Yes	Yes	Yes	Yes	Yes
Time fixed effect	Yes	Yes	Yes	Yes	Yes	Yes	Yes
Province fixed effect	Yes	Yes	Yes	Yes	Yes	Yes	Yes
*N*	9,345	3,424	578	7,470	5,877	2,411	10,936
*R* ^2^	0.114	0.079	0.282	0.081	0.123	0.091	0.131

① ^***^, ^**^, and ^*^ represent significance levels of 1, 5, and 10%, respectively. ② The numbers in parentheses are robust standard errors clustered at the provincial level.

#### 4.4.1 Skill level differences

Union membership exhibits a positive and statistically significant effect on employment across all skill groups. However, the effect size is notably larger for high-skilled workers compared to low-skilled and medium-skilled workers. This pattern suggests that unions may be more effective in enhancing employment outcomes for workers with higher education, possibly because they are more likely to be employed in formal sectors where union activities are more established and labor rights are more enforceable. In practical terms, union membership is associated with a 12.3 percentage-point increase in the probability of non-agricultural employment for high-skilled migrants, versus 8.9 and 7.0 percentage points for low- and medium-skilled groups, respectively.

#### 4.4.2 Gender differences

The positive effect of union membership is significant for both genders but is substantially stronger for female migrant workers than for males. This finding aligns with the notion that women face higher barriers in the labor market, including discrimination and informal employment arrangements. Unions may provide a more pronounced protective function for women by facilitating access to formal contracts, reducing wage gaps, and promoting workplace equity. The larger coefficient for women suggests that unionization could be a particularly effective policy lever for improving gender equity in migrant employment.

#### 4.4.3 Marital status differences

Unmarried migrant workers benefit more from union membership than their married counterparts. This may reflect differences in labor mobility and family constraints: unmarried workers are generally more geographically and occupationally mobile, and union support may amplify their ability to secure and maintain employment in urban labor markets. Married workers, often burdened with family responsibilities, may have less flexibility to leverage union resources for job transitions or upward mobility.

## 5 Mechanism analysis

The causal relationship established through the Treatment Effect Model confirms that union participation significantly enhances migrant workers’ likelihood of non-agricultural employment. Building upon the theoretical pathways outlined in Section 2, this section delves into the specific mechanisms through which unions exert their influence, directly links empirical findings to theoretical propositions, and situates the results within the broader scholarly conversation.

Our theoretical framework posited three interrelated pathways: (1) Labor rights protection and work-hour regulation, (2) Formalization of employment via labor contracts, and (3) Enhanced social security coverage fostering labor market attachment. The results presented in Table 6 provide robust empirical support for these hypothesized mechanisms and offer nuanced insights into how unions mitigate employment uncertainty for a vulnerable workforce.

**Table 6:** Mechanism test.

Variable	Income		Working hours		Labor contracts		Health insurance	
	(1)	(2)	(3)	(4)	(5)	(6)	(7)	(8)
	OLS	TEM-MLE	OLS	TEM-MLE	OLS	TEM-MLE	OLS	TEM-MLE
*Union*	0.194^***^ (0.046)	0.857^***^ (0.076)	−0.049^**^ (0.022)	−0.631^***^ (0.040)	0.673^***^ (0.142)	0.136^**^ (0.066)	0.491^***^ (0.160)	0.106^**^ (0.042)
Control variables	Yes	Yes	Yes	Yes	Yes	Yes	Yes	Yes
Time fixed effect	Yes	Yes	Yes	Yes	Yes	Yes	Yes	Yes
Province fixed effect	Yes	Yes	Yes	Yes	Yes	Yes	Yes	Yes
*N*	13,347	13,347	10,723	10,723	13,347	13,347	13,347	13,347

① ^***^, ^**^, and ^*^ represent significance levels of 1, 5, and 10%, respectively. ② The numbers in parentheses are robust standard errors clustered at the provincial level.

### 5.1 Empirical validation of theoretical pathways

First, regarding labor rights and work-hour regulation, our results show that union membership leads to a significant reduction in weekly working hours (Table 6, Columns 3–4). This aligns with collective bargaining theory (Freeman and Medoff, 1979) and recent findings that unions help curtail excessive overtime, a common issue for migrant workers (Xue and Li, 2022). Reduced work hours alleviate physical and psychological strain, which is critical for sustaining employment. As Sönmez et al. (2023) noted, excessive hours contribute to burnout and higher turnover. By promoting reasonable work schedules, unions enhance job sustainability, thereby directly contributing to employment stability—a core component of our theoretical model.

Second, concerning the formalization of employment relationships, we find a strong positive effect of union membership on the probability of having a written labor contract (Table 6, Columns 5–6). This finding resonates with employment protection theory (Ferreiro and Gomez, 2020) and empirical studies in China documenting unions’ role in improving contract coverage (Booth et al., 2022). A formal contract reduces ambiguity and arbitrariness in employment, providing a legal shield against unjust dismissal and wage arrears (Wang et al., 2019). This institutionalization of the employment relationship is a key mechanism through which unions reduce perceived job insecurity, thereby strengthening migrant workers’ attachment to their current employment and the formal labor market at large.

Third, pertaining to social security and labor market attachment, our analysis confirms that union membership significantly increases the probability of having health insurance (Table 6, Columns 7–8). This extends the findings of Aizawa et al. (2024) and Cheng and Wei (2024), highlighting unions’ role in bridging the social protection gap for migrant workers. Health insurance mitigates the financial shock of illness, reducing the risk of involuntary unemployment due to health crises (Wang et al., 2023). By lowering the economic vulnerability associated with health risks, unions enhance migrant workers’ long-term employability and their willingness to remain in urban labor markets. This mechanism underpins the broader concept of urban integration and labor market attachment theorized in Section 2.3.

### 5.2 Synthesis and connection to broader literature

Our mechanism analysis demonstrates that the employment effect of unions is not monolithic but operates through a synergistic combination of institutional formalization (contracts), risk mitigation (social insurance), and well-being enhancement (regulated hours). These empirical results collectively substantiate the theoretical proposition that unions function as vital institutional supports that reduce employment uncertainty.

Furthermore, our findings on labor contracts and social insurance provide empirical grounding for the urban integration pathway. Secure employment relations and social safety nets are foundational for migrant workers’ long-term urban settlement and social inclusion (Wang and Shen, 2022). While our primary focus and variable selection in Table 6 centered on proximal labor market outcomes (income, hours, contracts, insurance), these very factors are established precursors to broader socio-economic integration. Future research could incorporate direct measures of urban settlement intention or social network expansion to explicitly test this extended pathway.

The positive effects documented here are consistent with prior studies on unions’ protective roles in China (Ma, 2024; Ma and Komatsu, 2024) but extend the literature by explicitly linking these protective functions to employment stability outcomes rather than solely to wage levels. This addresses a gap noted by Sayre (2023), who called for more research on how institutional buffers affect employment-related insecurity. Our results suggest that in the Chinese context, unions partially offset the institutional disadvantages stemming from the hukou system by fostering more stable and protected employment conditions.

### 5.3 Limitations and implications for future research

While this study provides robust evidence supporting the positive association between union participation and migrant workers’ employment, several limitations should be acknowledged. First, despite our use of a treatment effect model and instrumental variables, endogeneity due to self-selection and unobserved heterogeneity cannot be fully ruled out. Union membership may still serve as a proxy for employment in more formalized sectors with better labor conditions. Second, our data are cross-sectional and lack longitudinal tracking, which limits our ability to examine the dynamic effects of union participation on employment stability over time. Third, the binary measure of union membership does not capture variations in union activity intensity or functional effectiveness, which may influence employment outcomes differently. Fourth, our mechanisms are inferred from observable outcomes (e.g., contracts, insurance) but lack direct measures of psychological constructs such as perceived job security or belongingness.

Future research could address these gaps by: (1) utilizing panel data or natural experiments (e.g., policy shocks in unionization campaigns) to strengthen causal identification; (2) incorporating mixed methods, including qualitative interviews, to explore how union participation shapes migrant workers’ psychological well-being and social integration; (3) developing more nuanced measures of union engagement and workplace institutionalization; and (4) examining the long-term effects of union participation on career progression and urban settlement.

## 6 Conclusion and policy recommendations

This study provides evidence consistent with a causal interpretation that union participation is associated with improved non-agricultural employment among migrant workers in China. Using a treatment effect model on nationally representative data, we find that this relationship operates through three plausible mechanisms: improved labor rights protection (reduced excessive working hours), formalization of employment relationships (increased written labor contracts), and enhanced social security coverage (higher health insurance enrollment). Heterogeneity analyses reveal that these benefits are particularly pronounced for high-skilled, female, and unmarried migrant workers.

The findings underscore the psychological and institutional roles of trade unions in mitigating employment uncertainty and strengthening labor market attachment—a critical dimension of migrant workers’ well-being and social integration. Building on these empirical insights, we propose the following concrete, actionable policy recommendations:

### 6.1 Strategically expand union coverage with a focus on vulnerable sectors and workers

Given the significant benefits identified, policymakers should prioritize expanding unionization in private enterprises, small and medium-sized firms, and labor-intensive industries where migrant workers are concentrated and formal protection is often weakest. Concrete actions could include: (a) simplifying union registration procedures for enterprises with a high proportion of migrant workers; (b) offering tax incentives or regulatory compliance benefits to firms that achieve high union membership rates; and (c) launching targeted campaigns to recruit female, low-skilled, and informal workers into union membership.

### 6.2 Institutionalize and monitor labor rights through digital platforms

To operationalize the mechanism of employment formalization, we recommend establishing a national digital labor contract and rights-monitoring platform integrated with social insurance systems. This platform should: (a) enable real-time, mandatory registration of all labor contracts, with automatic alerts for non-compliance or contract expiration; (b) provide a direct channel for workers to report violations of working-hour regulations or wage arrears, linked to union and labor inspection offices; and (c) publicly disclose enterprise compliance ratings to strengthen market and social supervision.

### 6.3 Enhance social insurance portability and accessibility via union-mediated schemes

To address the social security gap, unions should be empowered to administer or facilitate portable, subsidized social insurance packages tailored for migrant workers. Specific measures include: (a) piloting “union-managed health insurance pools” that allow seamless coverage across jobs and regions, with premium subsidies for low-income members; (b) mandating unions’ role in verifying and enrolling workers in existing urban insurance schemes; and (c) using union halls or mobile stations for on-site insurance enrollment and claims assistance.

### 6.4 Implement sector-specific working hour standards with union oversight

Building on our finding that unions reduce excessive hours, regulatory bodies should collaborate with unions to develop and enforce industry-specific working hour benchmarks. This involves: (a) tripartite negotiations (government, union, employer associations) to set realistic hourly limits per sector; (b) requiring unions to appoint “working-hour monitors” in large workplaces; and (c) introducing a mandatory rest-period log for high-intensity jobs, subject to random union audits.

### 6.5 Strengthen union capacity through targeted funding and training

To ensure unions can effectively deliver the mechanisms identified, state funding should support: (a) specialized training programs for union representatives on labor law, contract negotiation, and mental health support; (b) grants for unions to provide direct services such as legal aid clinics, vocational upskilling courses, and counseling hubs; and (c) performance-based funding tied to measurable outcomes in contract coverage, insurance enrollment, and grievance resolution for migrant workers.

These recommendations are directly derived from our empirical findings and are designed to translate the protective functions of unions into scalable, institutionalized practices. Future policy evaluations should assess the impact of these interventions not only on employment stability but also on psychological well-being and long-term urban integration of migrant workers.

## Data Availability

The data analyzed in this study is subject to the following licenses/restrictions: the processed dataset supporting the conclusions of this article is not publicly available due to privacy and confidentiality agreements related to the original CGSS survey, but can be made available from the corresponding author upon reasonable request for academic purposes. Requests to access these datasets should be directed to Hao Sun, 2073815881@qq.com.
